# Mapping county-level vulnerability to the energy transition in US fossil fuel communities

**DOI:** 10.1038/s41598-022-19927-6

**Published:** 2022-09-21

**Authors:** Daniel Raimi, Sanya Carley, David Konisky

**Affiliations:** 1 Resources for the Future, 1616 P St. NW, Suite 600, Washington, DC 20036 USA; 2 Paul H. O’Neill School of Public and Environmental Affairs, Indiana University, Bloomington, USA

**Keywords:** Climate-change impacts, Governance, Risk factors, Coal, Crude oil, Natural gas

## Abstract

The energy transition toward lower-carbon energy sources will inevitably result in socioeconomic impacts on certain communities, particularly those that have historically produced fossil fuel resources and electricity generation using fossil fuels. Such communities stand to lose jobs, tax revenues, and support for public services. Which communities are most likely to be affected, which are more susceptible to being harmed, and how to target adaptive capacity programs—such as economic development and workforce training—accordingly are pressing scholarly and policy questions. In this study, we apply a vulnerability framework to calculate, rank, and map exposure and sensitivity scores for fossil fuel producing regions in the US. We find that, while counties in most regions of the United States will be affected by the transition away from fossil fuels, counties in Appalachia, Texas and the Gulf Coast region, and the Intermountain West are likely to experience the most significant impacts, and some regions experience overlapping and significant incidence of vulnerability. These results can be used to target future adaptive capacity programs.

## Introduction

Numerous strategies exist to mitigate the urgent challenge of climate change, including emissions mitigation, negative emissions technologies, and geoengineering approaches^1–4^. To date, most efforts have centered on reducing emissions from the energy system, which is responsible for the large majority of anthropogenic greenhouse gas emissions. Indeed, limiting global warming to international targets necessitates an energy transition at unprecedented speed and scale^5^. As public policies coupled with innovation accelerate the deployment of clean energy and associated technologies, economic changes will lead to socioeconomic and environmental risks for specific communities across the US and the world^6–9^.

Although an energy transition away from fossil fuels will create new economic opportunities, it will also reduce employment and tax revenues from carbon-intensive activities^10–13^. Such trends are already evident in coal communities across the US, as 100 gigawatts (GW) of coal power plant capacity was retired between 2002 and 2021 and another 59 GW are planned for retirement by 2035^14^. The extant literature has documented that when coal and other facilities close, economic impacts extend well beyond direct job losses for facility employees^16^. Local and state jurisdictions often lose a significant portion of their tax revenues, which can affect schools and other public services^12,17–19^. In communities where fossil fuel extraction, processing, or centralized use (e.g., at power stations) is the main source of economic activity, there are typically spill-over effects on other industries such as on retail, food establishments, and construction^16,20–23^. Previous scholarship has also found significant social impacts: members of households may have to travel long distances for new employment opportunities; households may need to renegotiate their schedules and personal and professional circumstances; and the loss of employment may lead to a loss of a sense of community and culture^17,24,25^.

Certain mitigation technologies, such as carbon capture and storage (CCS) or biomass energy coupled with CCS (BECCS), could be implemented in some fossil energy communities^3,26^, which could allow for continued use of fossil fuels at scale. If these technologies are deployed at scale, they could dampen some of the local economic challenges associated with emissions mitigation by providing jobs and supporting tax revenues. However, deployment of these technologies has been limited to date, and there are concerns about the environmental and health impacts of both CCS and BECCS technologies^2,27,28^.

Although energy transitions are historically slow, since they require massive new infrastructure developments and widespread deployment of new technolgies^29–31^ and also significant behavioral adjustments^9,32^, the speed and scale of an energy transition consistent with international climate targets such as 1.5 °C or 2 °C will be unprecedented^5^. The impacts within communities, similarly, will be rapid, and will fall disproportionately on more vulnerable socio-economic groups, including those within fossil fuel communities. An important question—and one that is currently being asked by policymakers, practitioners, and scholars alike—is where such economic changes are likely to be concentrated, and which communities might be most vulnerable to disruptions?

Although achieving international climate targets will entail major reductions in extraction, processing, and use of all fossil fuels^26,33^, few studies have sought to identify with great precision which specific communities may be most impacted and most sensitive to such changes. While studies have generated projections about which power plants are most likely to shutter sooner rather than later^34^, few have looked across all fossil fuel industries in a comprehensive analysis. One notable exception is a study by Snyder^35^, which combines fossil fuel employment data from the US Bureau of Labor Statistics (BLS) with socioeconomic measures to create an index of energy transition vulnerability for US counties. The resulting metrics are aggregated across all industries, however, which obscures variation by industry and fuel type. As a result, for example, counties in Wyoming, which dominates US coal production, do not appear near the top of the index. Another exception is a study by Raimi^36^, which identifies the hundreds of US counties where fossil energy accounts for large shares of local employment and wages. This analysis, however, is incomplete because the underlying data are in some cases suppressed for low-population (typically rural) counties, which may be particularly vulnerable to the effects of the energy transition. In addition, the analysis did not incorporate measures of socioeconomic or environmental vulnerability, as we seek to do in the present study.

Here, we address these limitations in existing research and the challenging question about geographic vulnerability through a comprehensive approach. We do so by employing the vulnerability scoping framework first introduced by Polsky et al.^37^ and subsequently adapted by Carley et al.^38^ to the context of energy transition vulnerability. Specifically, we identify locations in the US that are most susceptible to economic losses associated with fossil fuels and combine this with socioeconomic measures of sensitivity within these communities. Using the vulnerablity framework, vulnerability is the net of three factors: exposure such as an economic shock (e.g., coal mine closure) or policy intervention (e.g., a carbon tax); sensitivity to this exposure (e.g., those who have few other economic opportunities); and adaptive capacity to respond to the exposure (e.g., local government resources that can support the community in transition). Carley et al.^38^ applied this framework to the context of energy price changes due to the energy transition, since the energy transition will likely exacerbate energy burdens as well^39^. In the present analysis, we adapt it to the context of fossil fuel communities, and the exposure that they face to declining economic activity. By combining measures of exposure and sensitivity, we produce an aggregate measure, distinguishing between fossil energy types (i.e., coal, oil, and natural gas) and activity (i.e., extraction, refining, and combustion at power stations), of which communities are especially vulnerable to the energy transition and need targeted adaptive capacity efforts.

The types of adaptive capacity that would be useful for an affected community includes local government resources and technical expertise, workforce skills, physical infrastructure, suitability for alternative economic development strategies, and other factors such as community cohesion. We do not attempt to incorporate these measures in our empirical analysis because, in many cases, they cannot be reliably measured and because it is unclear how to standardize across different metrics (e.g., workforce skills and community cohesion). As such, the measures we present here can be seen as identifying locations that may be most in need of enhanced adaptive capacity in the future.

Our analysis makes three primary scholarly contributions. First, we extend a useful conceptual framework to include the context of fossil fuel industry vulnerability to the US energy transition. Second, we contribute a methodology that other scholars can apply to produce similar results for other geographic contexts. Third, in so doing, we produce forward-looking results that can be used to anticipate future developments and target interventions accordingly. This is in contrast to previous applications of the vulnerability scoping metric that rely on past trends to estimate vulnerability^38^. Our analysis also serves a practical need. The resulting estimates and maps can be used by practitioners and policymakers as they identify communities that may be most in need of enhanced adaptive capacity to address the challenges posed by a transition away from fossil fuels.

## Results

### Quantitative results

**Figure 1:**
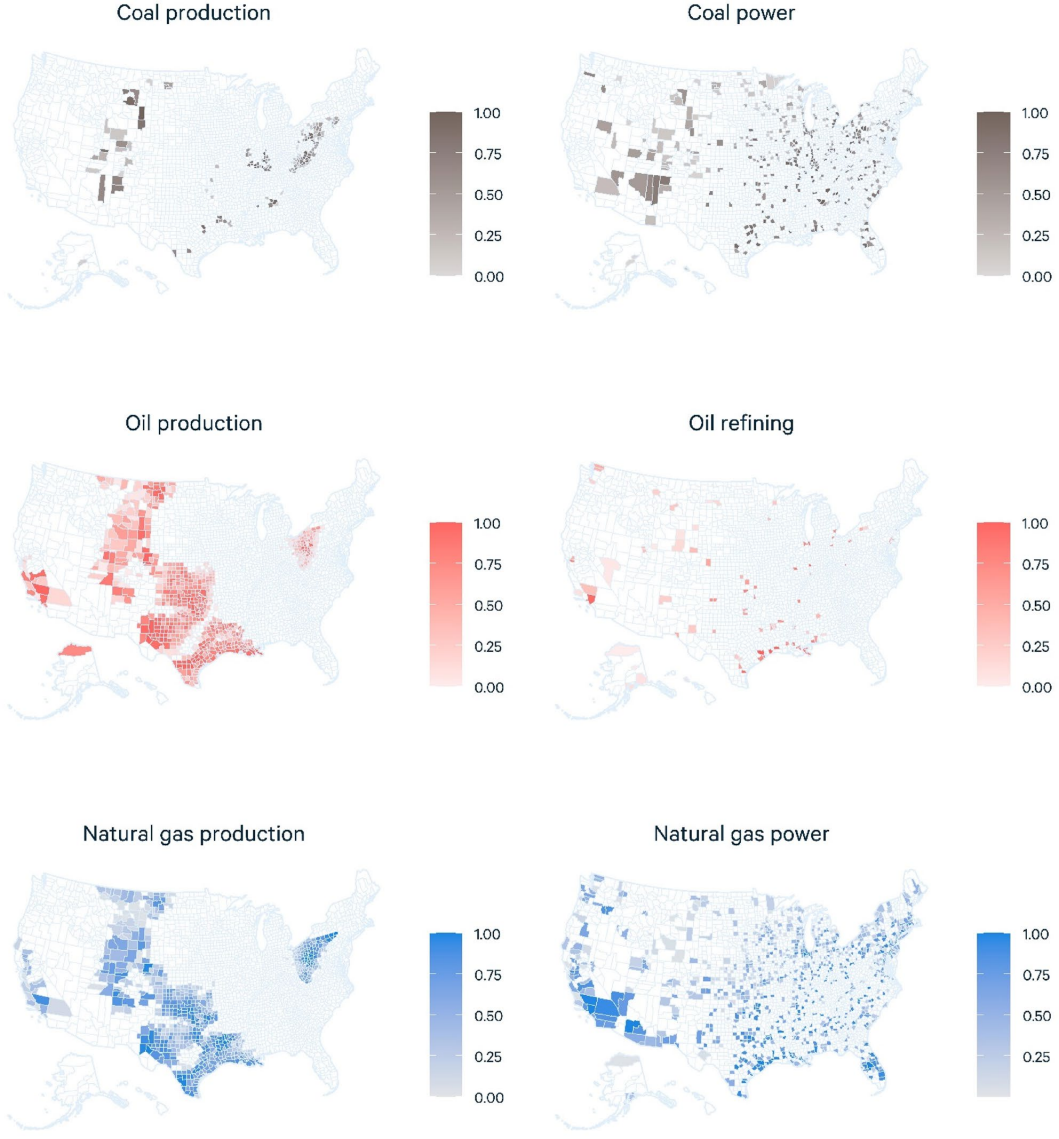
US county-level exposure and sensitivity to energy transition. These maps show results across all activity and fuel types, in which colored shading indicates the percentile of each US county’s exposure and sensitivity to a transition away from fossil fuels. Counties in the highest percentile for the combined measures of exposure and sensitivity are colored darkest, and counties with lower scores are colored more lightly.

Using the approach outlined in the “Methods” section, we identify the US counties with the highest combined exposure and sensitivity scores across six fossil fuel activities: coal extraction, coal-fired power, oil extraction, oil refining, natural gas extraction, and natural gas power. In Table 1 we present the highest 10 counties in each category; in the Supplemental Information, Tables SI1-6, we provide tables for each category in which we include the top 40 counties (full data are available in the data appendix), along with an aggregate score that combines all six metrics (Table SI8). In these tables, we present the final measures of exposure, sensitivity, and exposure multiplied by sensitivity, the latter in both raw and percentile form. In Fig. 1, we map the results across all activity and fuel types, in which colored shading indicates the percentile of each US county’s exposure and sensitivity to a transition away from fossil fuels. Counties in the highest percentile for the combined measures of exposure and sensitivity are colored darkest, and counties with lower scores are colored more lightly.

**Table 1:** Exposure, sensitivity, and exposure times sensitivity scores for the top 10 counties across all six fossil fuel categories.

County	Exposure	Sensitivity	Exposure * Sensitivity (raw score)	Exposure * Sensitivity (percentile)
**Coal production**				
Campbell County, WY	34.5	7	228	1.00
Marshall County, WV	2.6	83	216	0.99
Greene County, PA	4.0	48	193	0.99
Franklin County, IL	1.8	98	178	0.98
Union County, KY	1.6	96	154	0.97
Marion County, WV	1.8	82	151	0.97
Logan County, WV	1.6	92	150	0.96
Jefferson County, AL	1.3	91	114	0.95
Gibson County, IN	1.4	70	101	0.95
Limestone County, TX	1.4	72	97	0.94
**Coal-fired power production**				
Jefferson County, OH	1.3	91	123	1.00
Titus County, TX	1.1	91	99	1.00
Gallia County, OH	1.1	90	99	0.99
Person County, NC	1.0	93	92	0.99
Muhlenberg County, KY	0.8	95	80	0.99
Bartow County, GA	1.0	76	80	0.99
Jefferson County, AL	0.8	91	77	0.98
Monroe County, GA	1.1	70	75	0.98
Gibson County, IN	1.0	70	70	0.98
Indiana County, PA	1.4	47	67	0.98
**Oil production**				
Karnes County, TX	3.1	94	293	1.00
Reeves County, TX	3.4	48	162	1.00
Howard County, TX	2.5	59	150	1.00
Weld County, CO	4.9	30	146	1.00
Lea County, NM	5.5	26	144	0.99
Kern County, CA	3.2	43	139	0.99
La Salle County, TX	1.8	70	123	0.99
DeWitt County, TX	1.2	94	114	0.99
Gonzales County, TX	1.2	88	107	0.99
Midland County, TX	5.5	19	107	0.99
**Oil refining**				
Harris County, TX	8.5	95	803	1.00
Jefferson County, TX	8.1	92	745	0.99
Calcasieu Parish, LA	4.3	88	378	0.98
Los Angeles County, CA	5.5	68	372	0.97
St. John the Baptist Parish, LA	3.1	92	283	0.95
Nueces County, TX	4.4	58	251	0.94
East Baton Rouge Parish, LA	2.7	82	225	0.93
Galveston County, TX	4.3	51	219	0.92
Lake County, IN	2.3	86	197	0.91
Philadelphia County, PA	1.8	100	177	0.90
**Natural Gas Production**				
De Soto Parish, LA	3.2	87	276	1.00
Susquehanna County, PA	4.7	44	209	1.00
Belmont County, OH	2.6	76	201	1.00
Washington County, PA	3.3	48	159	1.00
Reeves County, TX	3.2	48	152	0.99
Monroe County, OH	1.6	94	151	0.99
Greene County, PA	2.9	48	139	0.99
Jefferson County, OH	1.5	91	138	0.99
Bradford County, PA	2.5	51	127	0.99
Webb County, TX	2.3	51	119	0.99
**Natural gas-fired power plants**				
Harris County, TX	1.5	95	144	1.00
Los Angeles County, CA	1.8	68	122	1.00
Heard County, GA	0.7	90	66	1.00
Queens County, NY	1.2	55	65	1.00
Maricopa County, AZ	2.1	27	57	1.00
St. Charles Parish, LA	0.8	76	57	0.99
Polk County, FL	1.0	56	54	0.99
Will County, IL	1.0	48	50	0.99
Northampton County, PA	0.7	73	48	0.99
Union County, AR	0.4	99	44	0.99

Notes: Exposure is reported on a scale of zero to 100, where 100 indicates that a given county accounts for 100 percent of the relevant fossil fuel activity (e.g., coal extraction) nationwide. Sensitivity scores are also reported on a scale of zero to 100, but differ from the exposure metrics by indicating each county’s percentile of all US counties based on the metrics for defining a “disadvantaged” community (see “Methods”). Aggregate scores are reported in two ways. The first presents the raw product of exposure times sensitivity, and the second ranks each county by percentile of all US counties with the relevant fossil fuel activity.

### Coal

Because it is the most carbon-intensive fuel, and because it has numerous substitutes in the power sector, coal will almost certainly be the energy source that declines most rapidly under ambitious climate policies^5,40^. Indeed, US coal production has declined dramatically in recent years, though most of this decline has been due to market factors led by low-cost natural gas^41^. Coal is produced mainly in the Intermountain West, Appalachia, and the Illinois basin, with limited production in other locations.

In 2019, more than one-third of all US coal was produced in Campbell County, Wyoming. However, Campbell County scores very low on our measures of sensitivity, indicating that it currently faces fewer environmental and socioeconomic challenges than most other US counties. Most other major coal-producing counties have considerably higher sensitivity scores, indicating relatively high rates of poverty and exposure to environmental burdens often found in parts of Appalachia and parts of the Intermountain West.

Hundreds of US coal-fired electric generating units have retired in recent decades or are planned for retirement, and absent widespread deployment of CCS, coal-fired generation will need to approach zero in the coming decades to achieve ambitious climate goals^5,26,40^. Because coal plants are widely distributed across the country, county-level exposure scores are lower than those seen in coal extraction, which is more geographically concentrated. However, sensitivity scores for these counties are very high, indicating high existing levels of poverty, low levels of education, and high exposure to environmental risks. As with coal production, exposure and sensitivity for coal power plant communities is most prevalent in Appalachia. However, numerous counties in regions with no coal production, such as Florida, North Carolina, and Wisconsin, also score highly on these measures.

### Oil

Oil is the US’ largest source of primary energy supply, and production has grown dramatically over roughly the past decade, led by parts of Texas, New Mexico, and North Dakota. However, substantial quantities of oil are produced in more than a dozen other states. In part because of the economic benefits of recent oil production growth, many of the counties with the highest level of exposure have low sensitivity scores. For example, McKenzie County, ND produced more oil than any other US county in 2019, but it only ranks in the 4th percentile for sensitivity.

The counties with the highest combined levels of exposure and sensitivity are concentrated in South and West Texas, where high levels of exposure are combined with relatively low income levels and high exposure to environmental and climate risks. For examples, Karnes County, in South Texas, ranks in the top 90th percentile for our climate change indicators and in the bottom 20th percentile for educational indicators. Although they do not rank in the top 10 counties listed in Table 1, there is also high exposure and sensitivity for certain counties in California, Louisiana, North Dakota, Utah, and Wyoming.

Oil refineries are essential components of today’s energy system, but they are also major polluters—of both greenhouse gases and air emissions that impose health burdens on nearby populations^42^. The United States is home to the largest fleet of oil refineries in the world^43^, centered along the Gulf Coast and near major population centers.

Reflecting the environmental burdens of refineries and other heavy industry that are often concentrated around major ports, most of the counties with high levels of exposure also have high levels of sensitivity. Indeed, the six counties with the highest combined exposure and sensitivity scores each host major port facilities, where crude oil, petroleum products, and other commodities are imported, refined, and exported in large quantities. In some counties (e.g., Will County, IL; Contra Costa County, CA) with high levels of refining, these environmental burdens are relatively low. However, our metrics are county-level aggregates, and may not reflect the more localized sensitivities that likely exist at smaller geographic scales (e.g., Census tracts or blocks).

### Natural gas

Natural gas is produced at scale in hundreds of US counties, and top producing counties are found in Pennsylvania, Louisiana, Texas, Colorado, Ohio, Wyoming, and New Mexico. Although it is less carbon intensive than coal or oil on an energy-equivalent basis (assuming methane emissions are minimized), US and global natural gas production declines in the coming decades under most ambitious climate scenarios^26^.

As with oil production, some of the most exposed US counties score relatively low on sensitivity metrics, in part because of the economic benefits of the growth in domestic natural gas production, and in part due to relatively low environmental burdens and climate change risks. For example, the top two natural gas producing counties in 2019, Susquehanna and Washington Counties, PA, rank in the bottom 50th percentile in our sensitivity score. However, other major producing counties in parts of Appalachia, Louisiana, and Texas show considerably higher sensitivity due to low economic indicators and high environmental and climate risk.

Like natural gas production, gas-fired power plants will tend to outlast coal because of their lower emissions intensity (if methane emissions are minimized). However, ambitious climate scenarios over the next several decades will require most gas-fired power to adopt CCS technologies, incorporate net-zero fuels such as biogas or hydrogen, or retire^26^. Existing capacity is concentrated in and around urban centers, allowing generators to respond quickly to increased demand during peak periods (e.g., hot summer days).

Many of these urban centers also show high sensitivity scores due to a variety of factors, including climate and other environmental risks (e.g., Harris County, TX); high housing and energy burdens (e.g., Los Angeles County, CA); and low levels of educational attainment coupled with poor public health indicators (e.g., Heard County, GA).

### Limitations

We believe this analysis provides the most comprehensive and policy-relevant information on which US communities could be affected by a transition away from fossil fuels. Our work, however, has several limitations that can be addressed through future research. First, we do not measure adaptive capacity, which will have considerable bearing on which communities are more resilient to shocks that reduce fossil fuel activities. Second, we do not attempt to identify the counties where fossil fuel activies would be more or less economically competitive in a scenario where national or global demand for fossil fuels decline. Third, we do not assess the role that negative emissions technologies, CCS, or other emerging technologies could play in extending the viability of fossil fuel extraction, processing, and use in some locations. Finally, we do not attempt to assess the importance or assign weights to different metrics in our construction of a vulnerability index. Future work could address these, and many other questions, to help enable an more equitible transition to a clean energy future.

## Discussion

Transitioning away from carbon-intensive energy sources is an urgent priority to limit the damages to society from climate change. However, this transition, especially at the pace required to meet international goals, will have concentrated costs for communities where fossil fuels are the current linchpin of local economic activity, employment, and public revenues. Some past research has sought to identify the communities most likely to be affected^35,36^, but extant work has relied on limited measures and often aggregates across fossil fuels, thereby obscuring important industry-specific patterns. We provide an aggregated score in the SI and discuss its shortcomings, particularly the difficulty in directly comparing between different fuels (i.e., coal, oil, and natural gas) and activities (i.e., extraction, processing, and use), along with some counterintuitive findings that result from such an approach. Our analysis addresses these limitations through a comprehensive accounting of counties in the United States that are most susceptible to economic losses associated with a downturn in coal, oil, and natural gas, as well as their intersections with socioeconomic indicators, building on the vulnerability scoping framework^37,38^.

There are several key findings. First, while all regions of the United States will be affected by the transition away from fossil fuels, counties in Appalachia, Texas and the Gulf Coast region, and the Intermountain West are likely to experience the most significant impacts. Second, the process of combining economic and socioeconomic indicators reveals a richer picture of these patterns, and underscores the importance of adopting a multi-facited vulnerability framework. An analysis focused solely on data measuring the volume of fossil fuel activities would not account for factors that could compound or ameliorate the economic impacts of declining fossil fuel production. Third, the industry-by-industry and fuel-by-fuel approach of our analysis shows that some counties are exposed to industry- or fuel-specific economic shocks or policy interventions, whereas others are exposed to shocks in multiple industries or fuels. It also shows that, although coal communities have been the focus of most policy intervention to date, a transition away from oil and natural gas would affect a much larger set of geographies and populations.

As noted earlier, an important limitation of our analysis is the omission of any measures of adaptive capacity. In the US, federal and state-level efforts are underway to enhance economic resilience and improve capacity for energy communities. For example, the federal Infrastrucuture Investment and Jobs Act of 2021 and the Inflation Reduction Act of 2022 provide financial incentives that encourage clean energy development in fossil energy communities^44,45^. Other federal programs, as well as state programs in California, Colorado, and New Mexico, are beginning to provide technical assistance to local governments to enhance their adaptive capacity^46–49^.

The results of our analysis can inform policymakers about which US counties are most vulnerable to the energy transition and where efforts can be directed to mitigate adverse impacts. For example, the US White House Interagency Working Group on Coal and Power Plant Communities and Economic Revitalization recently identified 25 US regions where fossil energy activities are concentrated, grouping regions by BLS metropolitan and nonmetropolitan classifications^46^. These groupings provide a useful starting point, but they are geographically coarse and lack consideration of socioeconomic and environmental factors.

The literatures on energy transitions and energy justice emphasize the importance of targeted interventions to support vulnerable communities^16,17,23,50–52^ and the need for these communities to build resilience both before and during the transition^53^. Yet, without a firm understanding of which communities are most vulnerable, it is challenging to identify which to target with compensatory measures. Vulnerability, as conceptualized in the framework we use in this analysis, is comprised of not just exposure and sensitivity, but adaptive capacity—the ability of communities to cope with or attenuate negative effects. Our analysis shows which communities should be targeted for adaptive capacity efforts, such as workforce (re)training programs, economic development initiatives, enhancing local government capacity, remediating polluted sites, and more.

The energy transition is both necessary and urgent for addressing climate change, and it is certain to bring with it economic opportunities, local environmental and climatic benefits, and technological innovations. The move away from fossil fuels, however, will also result in negative economic, social, and cultural impacts for many communities. For the energy transition to be “just,” it is important to both identify which communities are most likely to be affected, as we have done here, and to develop policies and programs to support those that experience negative impacts, a natural extension of this work.

## Methods

### Exposure

To develop a measure of exposure, we begin with energy data gathered from multiple sources to create a near-comprehensive county-level database of fossil fuel extraction, oil refining, and electricity production capacity in the United States. We do not include employment data because, as noted above, these data are often suppressed by the US Bureau of Labor Statistics to preserve confidentiality^36^. Coal extraction data come from the US Energy Information Administration (EIA)^54^, which in turn sources its data from the US Department of Labor’s Mine Safety and Health Administration. County-level oil and gas extraction datamust be aggregated from multiple state agencies and private data providers. For this analysis, we collected 2019 oil and natural gas production data from state agencies in Alaska, Arkansas, California, Colorado, Mississippi, Montana, North Dakota, New Mexico, Ohio, Pennsylvania, Texas, Utah, and Wyoming. Gathering county-level production data from state agencies for Kansas, Oklahoma, Louisiana, and West Virginia proved more challenging, so we use 2018 data (complete 2019 data were not available) collected and processed by Upton and Yu^55^. In 2020, these 17 states accounted for more than 99 percent of US onshore crude oil and natural gas production^56,57^. Oil refining and electricity capacity data are from the EIA^59^.

We focus on oil refining rather than oil-fired power generation because oil is used very little for power generation in the US. In addition, there is no equivalent for oil refining in coal and natural gas, which is typically more lightly processed than oil before its final consumption. Our exposure metric does not account for offshore oil and gas production, which provides substantial employment and public revenues, primarily along the Gulf Coast.

Our measure of exposure is useful but imperfect because it does not capture the economic, social, and political factors that may make some locations more resilient than others to a transition away from fossil fuels. For example, regions with lower-cost production (e.g., the Permian basin for oil) are likely to be more competitive than higher-cost producers (e.g., Kern County, California) in a scenario where demand for petroleum products declines. What’s more, state- and local-level policies will likely across geographies, creating environments where fossil fuel activities are encouraged in some states (e.g., Wyoming) and discouraged in others (e.g., California). Despite these limitations, our measure is useful and comprehensive.

### Sensitivity

To make this analysis as policy relevant as possible, we utilize a framework developed by the US federal government and released in April 2022 that identifies “vulnerable communities” using a range of metrics^60^. This framework, known as the Climate and Economic Justice Screening Tool (CEJST), was developed at the direction of federal Executive Order 14008, which instructs federal agencies to direct 40 percent of overall benefits of certain federal efforts towards “disadvantaged communities,” an initiative known as “Justice40”^61^. The tool includes 24 indicators grouped into eight categories. We adapt this framework into nine categories (explained below), shown in Table 2 (see SI for details).

**Table 2:** Climate and economic justice screening tool metrics. Source: White House Council on Environmental Quality^60^.

Category	Environmental/climate metrics
Climate change	Expected agriculture loss rate
	Expected building loss rate
	Expected population loss rate
Affordable and clean energy	Energy burden
	Particulate matter (PM) 2.5 concentration
Clean transit	Diesel particulate matter exposure
	Traffic proximity and volume
Affordable and sustainable housing	Percent of housing units built pre-1960
	Median home value
	Housing cost burden
Reduction and remediation of legacy pollution	Proximity to hazardous waste facilities
	Proximity to Risk Management Plan (RMP) facilities
	Proximity to National Priorities List (NPL or Superfund) sites
Critical clean water and wastewater infrastructure	Wastewater discharge
Health burdens	Asthma rates
	Diabetes rates
	Heart disease rates
	Life expectancy rates
Socioeconomic indicators	Poverty
	Linguistic isolation
	Unemployment
	Percentage of people living at or below 100% Federal poverty line
Education indicators	Educational attainment
	Higher education enrollment

The primary purpose of the CEJST is to develop a binary classification of Census tracts that are, or are not, “disadvantaged.” This is a logical approach in the context of allocating federal funding, where communities must be identified in a binary fashion for implementing Justice40. The proposed methodology considers a Census tract to be “disadvantaged” if it meets or exceeds a certain threshold—typically the 90th percentile of all tracts—for any single metric within a given category. For example, a tract would be classified as “disadvantaged” in the “health burdens” category if it exceeded the 90th percentile for asthma rates and also had low levels of income and higher education enrollment (see SI for details on the CEJST methodology).

For this analysis, we rely on the same metrics and categories as the CEJST but apply a different methodology. Instead of a binary classification, our main interest lies in identifying the range of sensitivities to future changes in fossil energy activities. As such, we use a continuous scale of zero to one, where zero is least sensitive and one is most sensitive. As noted above, the original CEJST tool includes eight categories that combine measures of environmental or climate metrics with socioeconomic metrics. This approach is useful in creating the binary classification system that is needed for Justice40. However, because our approach uses continuous measures, we separate the environmental and climate indicators from the socioeconomic indicators. We further adapt the CEJST by isolating educational metrics in a ninth category. We do so because decades of research has identified education as a necessary, though not necessarily sufficient, enabler of economic wellbeing^62,63^.

For each of the nine categories identified in Table 2, we take the following approach. First, we average the percentile of sensitivity for each metric (e.g., asthma, diabetes, heart disease, and life expectamcy rates) within each category (e.g., health burdens) for every Census tract. Next, we aggregate tracts to the county level, weighting each tract’s sensitivity score by population. We then take a simple average of the nine categories for each county. This approach effectively gives equal weight to all nine metrics. Although we considered weighting metrics to highlight certain sensitivity factors (e.g., energy or health burdens), we chose not to because we lack clear criteria for determining which metrics are more important than others.

Our final step uses this average to rank each county on a scale of 0–1, where 1 is the US county with the highest sensitivity score (Alexander County, IL) and 0 is county with the lowest sensitivity score (Teton County, WY).

### Combining exposure and sensitivity

Next, we combine our measures of exposure and sensitivity for every fossil fuel activity in every US county where such activity takes place. To do so, we multiply the levels of exposure and sensitivities in each county, following the methodology introduced in previous vulnerability score work^38^. For a measure of exposure, we use the aggregate level of fossil energy activity in the county rather than a standardized score. This approach provides additional weight to our exposure metric, where each county’s exposure score is equal to the nationwide proportion of fossil fuel activity (e.g., coal extraction) that occurs within the county. We believe such an approach is appropriate because the scales of fossil energy activity, and hence level of exposure, can vary dramatically even among top producers.

For example, Campbell County, WY produced more than eight times the amount of coal as the second largest producer (Greene County, PA) in 2019. Assigning a percentile rank for coal producing counties would therefore assign Campbell County, WY a score of 1 and Greene County, PA a score of 0.99, implying only a small difference between their exposure to a downturn in coal. In our view, such an approach would misrepresent the scale of exposure for two such counties and lead to a mischaracterization of the counties where the largest economic impacts are likely to be felt.

After multiplying the level of fossil energy activity by the sensitivity score, we then standardize the results on a scale of 0–1 for ease of interpretation, where 1 represents the county with the highest vulnerability and 0 represents a county with zero vulnerability.

## Supplementary Information

Supplementary Information 1.

Supplementary Information 2.

## Data Availability

All data analysed during the current study are included in this published article and its supplementary information files.
