# Supplementary material for: Mapping county-level vulnerability to the energy transition in US fossil fuel communities
Source: Sci Rep. 2022 Sep 21;12:15748. doi: 10.1038/s41598-022-19927-6 (PMC9492708; doi:10.1038/s41598-022-19927-6)
Supplement: Supplementary file 2 — Supplementary Information 2. [file 41598_2022_19927_MOESM2_ESM.docx]

Mapping County-Level Vulnerability to the Energy Transition in US Fossil Fuel Communities

Daniel Raimi^a^, Sanya Carley^b^, David Konisky^c^

^a^ Resources for the Future, [raimi@rff.org](mailto:raimi@rff.org)

^b^ Paul H. O’Neill School of Public and Environmental Affairs, Indiana University, [scarley@indiana.edu](mailto:scarley@indiana.edu)

^c^ Paul H. O’Neill School of Public and Environmental Affairs, Indiana University, [dkonisky@indiana.edu](mailto:dkonisky@indiana.edu)

Supplemental information

Supplemental Information

Detail on Climate and Economic Justice Screening Tool

CEJST metrics

Here, we provide additional information on the metrics used to calculate sensitivity scores for each US county. We rely on Census-tract level data released by the White House in April 2022.^1^ This dataset provides raw scores and an index that indicates each Census Tracts’ percentile of sensitivity among all tracts to each metric on a scale of 0-1. Those metrics, identified in Main Table 2, are:

*Expected agriculture loss rate*: Calculated by the CEJST based on data from the Federal Emergency Management Agency’s National Risk Index, 2014-2021.

*Expected building loss rate*: Calculated by the CEJST based on data from the Federal Emergency Management Agency’s National Risk Index, 2014-2021.

*Expected population loss rate*: Calculated by the CEJST based on data from the Federal Emergency Management Agency’s National Risk Index, 2014-2021.

*Energy burden*: Calculated by the CEJST based on data from the US Department of Housing and Urban Development’s Comprehensive Housing Affordability Strategy, 2014-2018.

*Particulate matter (PM) 2.5 concentration:* Data from the US Environmental Protection Agency’s EJScreen tool, 2014-2020.

*Diesel PM exposure*: Data from the US Environmental Protection Agency’s EJScreen tool, 2014-2020.

*Traffic proximity and volume:* Data from the US Environmental Protection Agency’s EJScreen tool, 2014-2020.

*Percent of housing units built pre-1960*: This is an indicator that lead paint may have been used within the home. Data from the US Census’ American Communities Survey, 2015-2019.

*Median home value*: Data from the US Census’ American Communities Survey, 2015-2019.

*Housing cost burden*: Calculated by the CEJST based on data from the US Department of Housing and Urban Development’s Comprehensive Housing Affordability Strategy, 2014-2018.

*Proximity to hazardous waste facilities*: Data from the US Environmental Protection Agency’s EJScreen tool, 2014-2020.

*Proximity to Risk Management Plan (RMP) facilities*: Data from the US Environmental Protection Agency’s EJScreen tool, 2014-2020.

*Proximity to National Priorities List (NPL or Superfund) sites:* Data from the US Environmental Protection Agency’s EJScreen tool, 2014-2020.

*Wastewater discharge:* Data from the US Environmental Protection Agency’s EJScreen tool, 2014-2020.

*Asthma rates*: Data from the US Center for Disease Control and Prevention’s PLACES: Local Data for Better Health data tool, 2016-2019.

*Diabetes rates:* Data from the US Center for Disease Control and Prevention’s PLACES: Local Data for Better Health data tool, 2016-2019.

*Heart disease rates:* Data from the US Center for Disease Control and Prevention’s PLACES: Local Data for Better Health data tool, 2016-2019.

*Life expectancy rates*: Data from the US Center for Disease Control and Prevention’s U.S. Small-area Life Extectancy Estimates Project, 2010-2015.

*Median income*: Data from the US Census’ American Communities Survey, 2015-2019.

*Linguistic isolation:* Calculated by the CEJST based on data from the US Census’ American Communities Survey, 2015-2019.

*Unemployment:* Data from the US Census’ American Communities Survey, 2015-2019.

*Percentage of people living at or below 100% Federal poverty line*: Calculated by the CEJST based on data from the US Census’ American Communities Survey, 2015-2019.

*Educational attainment*: Estimate of high school degree attainment calculated by the CEJST based on data from the US Census’ American Communities Survey, 2015-2019.

*Higher education enrollment*: Data from the US Census’ American Communities Survey, 2015-2019.

Methodology for estimating county-level sensitivity

Here, we provide additional information on how we utilize the metrics described above to calculate a sensitivity score for each US county, and how that approach differs from the one used by the CEJST. In the CEJST approach, Census tracts are typically defined as “disadvantaged” if they satisfy two criteria:

1. Score at or above the 90^th^ percentile of Census tracts in any single metric, and
2. Is classified as a low-income area with low enrollment in higher education. The rationale here is that some communities are low-income because a large proportion of the population is enrolled in higher education, rather than having low-income because of poor economic conditions.

In our analysis, we do not seek to classify counties (or Census tracts) as either disadvantaged or not. Instead, we are interested in which locations are more or less sensitive to the variables used in the CEJST analysis. We therefore rely on percentile rankings provided by the CEJST data for each metric in each tract. For each of the nine categories described in Column 1 of Main Table 2, we create a percentile score for each tract by taking a simple average of each metric identified in Column 2 of Main Table 2. For example, a tract’s percentile ranking for sensitivity to climate change is calculated as an average of that tract’s percentile ranking for expected agriculture, building, and population loss rate.

Next, we aggregate tract-level data to the county level. This aggregation is necessary because much of the fossil fuel activity data (particularly oil and natural gas production) is only available at the county level. As noted in the main text, we aggregate to the county level by weighting each Census tract by population. This produces a percentile ranking for every US county for each of the nine categories identified in Column 1 ofTables SI1 through SI6. To create an aggregate sensitivity score for each county, we again take a simple average of these nine categories.

Table SI1 Most Exposed and Sensitive US Coal-Producing Counties

| County | Exposure | Sensitivity | Exposure * Sensitivity (raw score) | Exposure * Sensitivity (percentile) |
| --- | --- | --- | --- | --- |
| Campbell County, WY | 34.5 | 7 | 228 | 1.00 |
| Marshall County, WV | 2.6 | 83 | 216 | 0.99 |
| Greene County, PA | 4.0 | 48 | 193 | 0.99 |
| Franklin County, IL | 1.8 | 98 | 178 | 0.98 |
| Union County, KY | 1.6 | 96 | 154 | 0.97 |
| Marion County, WV | 1.8 | 82 | 151 | 0.97 |
| Logan County, WV | 1.6 | 92 | 150 | 0.96 |
| Jefferson County, AL | 1.3 | 91 | 114 | 0.95 |
| Gibson County, IN | 1.4 | 70 | 101 | 0.95 |
| Limestone County, TX | 1.4 | 72 | 97 | 0.94 |
| Sullivan County, IN | 1.2 | 81 | 95 | 0.93 |
| Big Horn County, MT | 2.6 | 35 | 92 | 0.93 |
| Knox County, IN | 0.9 | 97 | 89 | 0.92 |
| Ohio County, WV | 1.0 | 73 | 76 | 0.91 |
| Kanawha County, WV | 0.8 | 96 | 76 | 0.91 |
| Raleigh County, WV | 0.9 | 74 | 70 | 0.90 |
| Washington County, PA | 1.4 | 48 | 68 | 0.89 |
| Williamson County, IL | 0.7 | 88 | 65 | 0.89 |
| Monroe County, OH | 0.7 | 94 | 63 | 0.88 |
| Mingo County, WV | 0.7 | 94 | 63 | 0.87 |
| Carbon County, UT | 1.1 | 53 | 60 | 0.86 |
| Perry County, IL | 0.6 | 93 | 59 | 0.86 |
| Buchanan County, VA | 0.9 | 60 | 57 | 0.85 |
| Wyoming County, WV | 0.7 | 78 | 54 | 0.84 |
| Converse County, WY | 3.3 | 16 | 53 | 0.84 |
| Mcdowell County, WV | 0.5 | 98 | 52 | 0.83 |
| Washington County, IL | 0.9 | 51 | 47 | 0.82 |
| Hopkins County, KY | 0.5 | 88 | 46 | 0.82 |
| Boone County, WV | 0.6 | 73 | 44 | 0.81 |
| Saline County, IL | 0.4 | 96 | 40 | 0.80 |
| Taylor County, WV | 0.6 | 63 | 38 | 0.80 |
| Randolph County, IL | 0.5 | 83 | 38 | 0.79 |
| Hamilton County, IL | 0.8 | 44 | 37 | 0.78 |
| Musselshell County, MT | 1.0 | 36 | 36 | 0.78 |
| Harrison County, TX | 0.5 | 74 | 35 | 0.77 |
| Walker County, AL | 0.4 | 91 | 34 | 0.76 |
| Choctaw County, MS | 0.4 | 88 | 34 | 0.76 |
| Fayette County, WV | 0.4 | 88 | 33 | 0.75 |
| Pike County, KY | 0.4 | 81 | 33 | 0.74 |
| Perry County, KY | 0.4 | 84 | 32 | 0.74 |

Table SI2. Most Exposed and Sensitive US Coal-Fired Power Plant Counties

| County | Exposure | Sensitivity | Exposure * Sensitivity (raw score) | Exposure * Sensitivity (percentile) |
| --- | --- | --- | --- | --- |
| Jefferson County, OH | 1.3 | 91 | 123 | 1.00 |
| Titus County, TX | 1.1 | 91 | 99 | 1.00 |
| Gallia County, OH | 1.1 | 90 | 99 | 0.99 |
| Person County, NC | 1.0 | 93 | 92 | 0.99 |
| Muhlenberg County, KY | 0.8 | 95 | 80 | 0.99 |
| Bartow County, GA | 1.0 | 76 | 80 | 0.99 |
| Jefferson County, AL | 0.8 | 91 | 77 | 0.98 |
| Monroe County, GA | 1.1 | 70 | 75 | 0.98 |
| Gibson County, IN | 1.0 | 70 | 70 | 0.98 |
| Indiana County, PA | 1.4 | 47 | 67 | 0.98 |
| Carroll County, KY | 0.7 | 100 | 66 | 0.97 |
| Mason County, WV | 0.7 | 92 | 66 | 0.97 |
| Beaver County, PA | 0.8 | 79 | 65 | 0.97 |
| Franklin County, MO | 0.7 | 86 | 62 | 0.97 |
| Milwaukee County, WI | 0.8 | 75 | 60 | 0.96 |
| Marshall County, WV | 0.7 | 83 | 59 | 0.96 |
| Coshocton County, OH | 0.7 | 88 | 57 | 0.96 |
| Jefferson County, KY | 0.7 | 80 | 57 | 0.96 |
| Stewart County, TN | 0.8 | 71 | 56 | 0.95 |
| Pike County, IN | 0.7 | 78 | 55 | 0.95 |
| Harrison County, WV | 0.8 | 81 | 67 | 0.93 |
| Jefferson County, AR | 0.7 | 90 | 65 | 0.93 |
| Randolph County, IL | 0.8 | 83 | 63 | 0.93 |
| Robertson County, TX | 0.9 | 71 | 61 | 0.92 |
| McCracken County, KY | 0.6 | 96 | 61 | 0.92 |
| Catawba County, NC | 0.9 | 71 | 61 | 0.92 |
| St. Clair County, IL | 0.7 | 85 | 60 | 0.91 |
| Jefferson County, KY | 0.7 | 80 | 55 | 0.91 |
| Marshall County, WV | 0.7 | 83 | 55 | 0.90 |
| Mason County, KY | 0.6 | 85 | 55 | 0.90 |
| Bexar County, TX | 1.0 | 57 | 54 | 0.90 |
| Limestone County, TX | 0.7 | 72 | 53 | 0.89 |
| Trimble County, KY | 0.6 | 94 | 53 | 0.89 |
| Cleveland County, NC | 0.6 | 85 | 53 | 0.88 |
| New Madrid County, MO | 0.5 | 100 | 52 | 0.88 |
| Fort Bend County, TX | 1.1 | 47 | 52 | 0.88 |
| Montour County, PA | 0.7 | 72 | 51 | 0.87 |
| Roane County, TN | 0.7 | 72 | 49 | 0.87 |
| Putnam County, FL | 0.6 | 83 | 49 | 0.86 |
| Pleasants County, WV | 0.6 | 88 | 49 | 0.86 |

Note: Exposure metrics includes operating and retired power plants.

TableSI3. Most Exposed and Sensitive US Oil–Producing Counties

| County | Exposure | Sensitivity | Exposure * Sensitivity (raw score) | Exposure * Sensitivity (percentile) |
| --- | --- | --- | --- | --- |
| Karnes County, TX | 3.1 | 94 | 293 | 1.00 |
| Reeves County, TX | 3.4 | 48 | 162 | 1.00 |
| Howard County, TX | 2.5 | 59 | 150 | 1.00 |
| Weld County, CO | 4.9 | 30 | 146 | 1.00 |
| Lea County, NM | 5.5 | 26 | 144 | 0.99 |
| Kern County, CA | 3.2 | 43 | 139 | 0.99 |
| La Salle County, TX | 1.8 | 70 | 123 | 0.99 |
| DeWitt County, TX | 1.2 | 94 | 114 | 0.99 |
| Gonzales County, TX | 1.2 | 88 | 107 | 0.99 |
| Midland County, TX | 5.5 | 19 | 107 | 0.99 |
| Dimmit County, TX | 1.2 | 84 | 100 | 0.98 |
| Martin County, TX | 3.9 | 20 | 79 | 0.98 |
| Loving County, TX | 2.4 | 27 | 65 | 0.98 |
| Kingfisher County, OK | 1.1 | 50 | 56 | 0.98 |
| Upton County, TX | 2.2 | 23 | 49 | 0.98 |
| Eddy County, NM | 3.9 | 11 | 43 | 0.98 |
| Atascosa County, TX | 0.7 | 53 | 39 | 0.98 |
| Ward County, TX | 1.4 | 25 | 36 | 0.97 |
| Blaine County, OK | 0.6 | 50 | 32 | 0.97 |
| Guernsey County, OH | 0.4 | 82 | 32 | 0.97 |
| Mcmullen County, TX | 0.9 | 36 | 32 | 0.97 |
| Gaines County, TX | 0.7 | 45 | 31 | 0.97 |
| Burleson County, TX | 0.5 | 64 | 30 | 0.97 |
| Grady County, OK | 0.6 | 47 | 30 | 0.97 |
| Plaquemines Parish, LA | 0.3 | 83 | 27 | 0.96 |
| Reagan County, TX | 1.5 | 17 | 26 | 0.96 |
| Live Oak County, TX | 0.4 | 70 | 25 | 0.96 |
| Scurry County, TX | 0.5 | 53 | 24 | 0.96 |
| Los Angeles County, CA | 0.3 | 68 | 23 | 0.96 |
| Yoakum County, TX | 0.8 | 29 | 23 | 0.96 |
| Glasscock County, TX | 1.5 | 15 | 23 | 0.95 |
| McKenzie County, ND | 6.0 | 4 | 23 | 0.95 |
| Ector County, TX | 0.6 | 38 | 23 | 0.95 |
| Winkler County, TX | 0.6 | 33 | 21 | 0.95 |
| Zavala County, TX | 0.2 | 97 | 20 | 0.95 |
| Stephens County, OK | 0.2 | 84 | 19 | 0.95 |
| Hockley County, TX | 0.3 | 55 | 19 | 0.95 |
| Pecos County, TX | 1.0 | 18 | 19 | 0.94 |
| Andrews County, TX | 1.1 | 16 | 19 | 0.94 |
| Garvin County, OK | 0.3 | 65 | 18 | 0.94 |

Table SI4. Most Exposed and Sensitive US Oil-Refining Counties

| County | Exposure | Sensitivity | Exposure * Sensitivity (raw score) | Exposure * Sensitivity (percentile) |
| --- | --- | --- | --- | --- |
| Harris County, TX | 8.5 | 95 | 803 | 1.00 |
| Jefferson County, TX | 8.1 | 92 | 745 | 0.99 |
| Calcasieu Parish, LA | 4.3 | 88 | 378 | 0.98 |
| Los Angeles County, CA | 5.5 | 68 | 372 | 0.97 |
| St. John the Baptist Parish, LA | 3.1 | 92 | 283 | 0.95 |
| Nueces County, TX | 4.4 | 58 | 251 | 0.94 |
| East Baton Rouge Parish, LA | 2.7 | 82 | 225 | 0.93 |
| Galveston County, TX | 4.3 | 51 | 219 | 0.92 |
| Lake County, IN | 2.3 | 86 | 197 | 0.91 |
| Philadelphia County, PA | 1.8 | 100 | 177 | 0.90 |
| St. Charles Parish, LA | 2.3 | 76 | 177 | 0.89 |
| St. Bernard Parish, LA | 1.7 | 100 | 166 | 0.87 |
| Madison County, IL | 1.9 | 75 | 141 | 0.86 |
| Boyd County, KY | 1.5 | 92 | 141 | 0.85 |
| Crawford County, IL | 1.3 | 96 | 129 | 0.84 |
| Lucas County, OH | 1.7 | 74 | 128 | 0.83 |
| Jackson County, MS | 1.9 | 62 | 116 | 0.82 |
| Plaquemines Parish, LA | 1.4 | 83 | 112 | 0.80 |
| Will County, IL | 2.2 | 48 | 107 | 0.79 |
| Contra Costa County, CA | 3.6 | 28 | 102 | 0.78 |
| Union County, NJ | 1.4 | 70 | 96 | 0.76 |
| St. James Parish, LA | 1.1 | 83 | 92 | 0.75 |
| Brazoria County, TX | 1.4 | 53 | 74 | 0.74 |
| Shelby County, TN | 1.0 | 75 | 72 | 0.72 |
| Wayne County, MI | 0.7 | 92 | 68 | 0.71 |
| Delaware County, PA | 1.0 | 67 | 67 | 0.70 |
| Moore County, TX | 1.0 | 60 | 62 | 0.69 |
| Allen County, OH | 0.9 | 63 | 59 | 0.68 |
| Tulsa County, OK | 0.8 | 72 | 59 | 0.67 |
| New Castle County, DE | 1.0 | 56 | 54 | 0.66 |
| Hutchinson County, TX | 0.8 | 68 | 53 | 0.64 |
| Montgomery County, KS | 0.7 | 72 | 50 | 0.63 |
| Union County, AR | 0.5 | 99 | 48 | 0.62 |
| St. Landry Parish, LA | 0.4 | 99 | 42 | 0.61 |
| West Baton Rouge Parish, LA | 0.4 | 94 | 37 | 0.60 |
| Butler County, KS | 0.9 | 43 | 37 | 0.59 |
| Mobile County, AL | 0.5 | 80 | 37 | 0.57 |
| Stark County, OH | 0.5 | 71 | 36 | 0.56 |

Table SI5. Most Exposed and Sensitive US Natural Gas-Producing Counties

|  | Exposure | Sensitivity | Exposure * Sensitivity (raw score) | Exposure * Sensitivity (percentile) |
| --- | --- | --- | --- | --- |
| De Soto Parish, LA | 3.2 | 87 | 276 | 1.00 |
| Susquehanna County, PA | 4.7 | 44 | 209 | 1.00 |
| Belmont County, OH | 2.6 | 76 | 201 | 1.00 |
| Washington County, PA | 3.3 | 48 | 159 | 1.00 |
| Reeves County, TX | 3.2 | 48 | 152 | 0.99 |
| Monroe County, OH | 1.6 | 94 | 151 | 0.99 |
| Greene County, PA | 2.9 | 48 | 139 | 0.99 |
| Jefferson County, OH | 1.5 | 91 | 138 | 0.99 |
| Bradford County, PA | 2.5 | 51 | 127 | 0.99 |
| Webb County, TX | 2.3 | 51 | 119 | 0.99 |
| Karnes County, TX | 1.1 | 94 | 99 | 0.98 |
| Caddo Parish, LA | 1.0 | 96 | 98 | 0.98 |
| Red River Parish, LA | 1.0 | 91 | 93 | 0.98 |
| Panola County, TX | 1.3 | 69 | 87 | 0.98 |
| Weld County, CO | 2.7 | 30 | 81 | 0.98 |
| DeWitt County, TX | 0.8 | 94 | 72 | 0.98 |
| Culberson County, TX | 1.1 | 68 | 72 | 0.98 |
| Lycoming County, PA | 1.1 | 64 | 68 | 0.97 |
| Dimmit County, TX | 0.8 | 84 | 67 | 0.97 |
| Tyler County, WV | 0.8 | 79 | 62 | 0.97 |
| Tarrant County, TX | 1.1 | 56 | 61 | 0.97 |
| San Augustine County, TX | 0.6 | 89 | 58 | 0.97 |
| La Salle County, TX | 0.8 | 70 | 57 | 0.97 |
| Wetzel County, WV | 0.6 | 96 | 54 | 0.97 |
| Blaine County, OK | 1.0 | 50 | 51 | 0.96 |
| Lincoln Parish, LA | 0.7 | 66 | 49 | 0.96 |
| Doddridge County, WV | 1.2 | 38 | 47 | 0.96 |
| Bossier Parish, LA | 0.6 | 75 | 44 | 0.96 |
| Wyoming County, PA | 0.9 | 47 | 42 | 0.96 |
| Grady County, OK | 0.9 | 47 | 41 | 0.96 |
| Lea County, NM | 1.5 | 26 | 40 | 0.95 |
| Harrison County, TX | 0.5 | 74 | 40 | 0.95 |
| Marshall County, WV | 0.5 | 83 | 39 | 0.95 |
| Pittsburg County, OK | 0.5 | 81 | 38 | 0.95 |
| Ritchie County, WV | 0.6 | 66 | 38 | 0.95 |
| Harrison County, OH | 0.6 | 58 | 35 | 0.95 |
| Stephens County, OK | 0.4 | 84 | 32 | 0.95 |
| Loving County, TX | 1.2 | 27 | 32 | 0.94 |
| Hughes County, OK | 0.4 | 82 | 29 | 0.94 |
| Harrison County, WV | 0.4 | 81 | 28 | 0.94 |

TableSI6. Most Exposed and Sensitive US Natural Gas-Fired Power Plant Counties

| County | Exposure | Sensitivity | Exposure * Sensitivity (raw score) | Exposure * Sensitivity (percentile) |
| --- | --- | --- | --- | --- |
| Harris County, TX | 1.5 | 95 | 144 | 1.00 |
| Los Angeles County, CA | 1.8 | 68 | 122 | 1.00 |
| Heard County, GA | 0.7 | 90 | 66 | 1.00 |
| Queens County, NY | 1.2 | 55 | 65 | 1.00 |
| Maricopa County, AZ | 2.1 | 27 | 57 | 1.00 |
| St. Charles Parish, LA | 0.8 | 76 | 57 | 0.99 |
| Polk County, FL | 1.0 | 56 | 54 | 0.99 |
| Will County, IL | 1.0 | 48 | 50 | 0.99 |
| Northampton County, PA | 0.7 | 73 | 48 | 0.99 |
| Union County, AR | 0.4 | 99 | 44 | 0.99 |
| Richmond County, NC | 0.5 | 91 | 43 | 0.99 |
| Iberville Parish, LA | 0.4 | 99 | 42 | 0.99 |
| Chambers County, TX | 0.6 | 70 | 39 | 0.99 |
| Kern County, CA | 0.9 | 43 | 39 | 0.98 |
| Orange County, TX | 0.5 | 84 | 39 | 0.98 |
| Haywood County, TN | 0.4 | 95 | 36 | 0.98 |
| Union County, NJ | 0.5 | 70 | 36 | 0.98 |
| Jefferson Parish, LA | 0.4 | 78 | 35 | 0.98 |
| Mayes County, OK | 0.4 | 84 | 34 | 0.98 |
| Cherokee County, SC | 0.3 | 98 | 34 | 0.98 |
| Butler County, OH | 0.4 | 83 | 33 | 0.98 |
| Hardee County, FL | 0.4 | 81 | 31 | 0.98 |
| Tulsa County, OK | 0.4 | 72 | 31 | 0.97 |
| Calcasieu Parish, LA | 0.4 | 88 | 31 | 0.97 |
| Hot Spring County, AR | 0.4 | 83 | 31 | 0.97 |
| Mobile County, AL | 0.4 | 80 | 30 | 0.97 |
| Bexar County, TX | 0.5 | 57 | 30 | 0.97 |
| Wharton County, TX | 0.4 | 84 | 29 | 0.97 |
| Dallas County, TX | 0.3 | 88 | 29 | 0.97 |
| Choctaw County, MS | 0.3 | 88 | 28 | 0.97 |
| Middlesex County, NJ | 0.5 | 51 | 28 | 0.96 |
| Ouachita Parish, LA | 0.4 | 73 | 27 | 0.96 |
| Delaware County, PA | 0.4 | 67 | 27 | 0.96 |
| Oklahoma County, OK | 0.5 | 57 | 27 | 0.96 |
| Nueces County, TX | 0.5 | 58 | 27 | 0.96 |
| Morgan County, AL | 0.3 | 81 | 27 | 0.96 |
| Providence County, RI | 0.3 | 84 | 26 | 0.96 |
| Lawrence County, OH | 0.3 | 99 | 26 | 0.96 |
| Washington County, OH | 0.3 | 84 | 25 | 0.96 |
| Acadia Parish, LA | 0.3 | 76 | 25 | 0.95 |

Aggregate scores by county

As discussed in our main analysis, a key contribution of this work is to provide information about county-level exposure and sensitivity that disaggregates different fuel types. However, some researchers and policymakers may also be interested in the aggregate score across the six fossil fuel activities we quantify. Here, we present such an aggregate and discuss its merits and shortcomings.

Our first aggregate measure is a simple summation of the six measures shown in Tables SI1 through SI6. The main advantage of this approach is its simplicity. However, a major flaw is that a simple summation ignores the distinction between fuels. As discussed in the main text, certain fuels like coal are likely to be phased out more quickly than others such as natural gas in an energy transition.

To address this concern, we present an alternative aggregate score that weights each indicator by the carbon-intensity of the fuel source. This approach provides a rough indication of which fuels are more or less likely to be affected by efforts to reduce greenhouse gas emissions in the energy system. We weight each measure of exposure * sensitivity using estimates of the CO_2_ emissions from the combustion of coal, oil, and natural gas. Because coals, oils, and natural gases vary in their emissions intensity, we use the most commonly consumed form of each fuel: subbituminous for coal, gasoline for oil, and methane for natural gas. Table SI7 provides information on the CO_2_ content of each fuel and the resulting weights, which are calculated by dividing each fuel by the most carbon intensive fuel (coal). Note that these metrics exclude methane and other non-combustion emissions, which can add considerably to the lifecycle greenhouse gas footprints of all three fuels.

Table SI7 Weights used to assess carbon intensity of fuels

| Fossil fuel | Final product consumed | Kg CO_2_ per MMBtu | Weight |
| --- | --- | --- | --- |
| Coal | Bittuminous coal | 97.13 | 1.00 |
| Oil | Motor gasoline | 70.66 | 0.73 |
| Natural gas | Methane | 52.91 | 0.54 |

Data source: U.S. Energy Information Administration.^2^

Table SI8 presents the 40 counties that score highest in our weighted aggregate measure, alongside an unweighted aggregate and scores for each fossil fuel activity.

Table SI8 Aggregate sensitivity and exposure scores across all fossil energy activities

| County | Coal prod. | Coal power | Oil prod. | Oil refining | NG prod. | NG power | Aggregete (unweighted) | Aggregate (weighted) |
| --- | --- | --- | --- | --- | --- | --- | --- | --- |
| Marshall County, WV | 0.99 | 0.96 | 0.91 | 0.00 | 0.95 | 0.50 | 4.32 | 3.41 |
| Rusk County, TX | 0.70 | 0.95 | 0.83 | 0.00 | 0.92 | 0.84 | 4.24 | 3.21 |
| Harrison County, TX | 0.77 | 0.70 | 0.68 | 0.00 | 0.95 | 0.86 | 3.96 | 2.95 |
| Calcasieu Parish, LA | 0.00 | 0.70 | 0.81 | 0.98 | 0.64 | 0.97 | 4.10 | 2.88 |
| San Juan County, NM | 0.68 | 0.80 | 0.82 | 0.00 | 0.91 | 0.29 | 3.51 | 2.73 |
| Robertson County, TX | 0.66 | 0.93 | 0.86 | 0.00 | 0.88 | 0.00 | 3.33 | 2.69 |
| Washington County, PA | 0.89 | 0.60 | 0.90 | 0.00 | 1.00 | 0.00 | 3.39 | 2.69 |
| Limestone County, TX | 0.94 | 0.90 | 0.35 | 0.00 | 0.83 | 0.00 | 3.02 | 2.55 |
| De Soto Parish, LA | 0.61 | 0.73 | 0.56 | 0.00 | 1.00 | 0.42 | 3.33 | 2.53 |
| Greene County, PA | 0.99 | 0.79 | 0.23 | 0.00 | 0.99 | 0.00 | 3.00 | 2.49 |
| Campbell County, WY | 1.00 | 0.31 | 0.86 | 0.00 | 0.65 | 0.22 | 3.05 | 2.41 |
| Converse County, WY | 0.84 | 0.41 | 0.94 | 0.00 | 0.80 | 0.00 | 2.99 | 2.36 |
| Armstrong County, PA | 0.42 | 0.91 | 0.14 | 0.00 | 0.88 | 0.74 | 3.09 | 2.31 |
| Atascosa County, TX | 0.68 | 0.48 | 0.98 | 0.00 | 0.78 | 0.00 | 2.91 | 2.29 |
| Los Angeles County, CA | 0.00 | 0.00 | 0.96 | 0.97 | 0.61 | 1.00 | 3.53 | 2.27 |
| Harris County, TX | 0.00 | 0.00 | 0.79 | 1.00 | 0.69 | 1.00 | 3.48 | 2.22 |
| Kanawha County, WV | 0.91 | 0.63 | 0.36 | 0.00 | 0.76 | 0.00 | 2.65 | 2.21 |
| Muhlenberg County, KY | 0.71 | 0.99 | 0.00 | 0.00 | 0.00 | 0.91 | 2.61 | 2.19 |
| Pointe Coupee Parish, LA | 0.00 | 0.87 | 0.66 | 0.00 | 0.64 | 0.90 | 3.08 | 2.19 |
| Fort Bend County, TX | 0.00 | 0.89 | 0.69 | 0.00 | 0.58 | 0.89 | 3.05 | 2.19 |
| Fayette County, TX | 0.00 | 0.83 | 0.82 | 0.00 | 0.78 | 0.55 | 2.98 | 2.15 |
| Knox County, IN | 0.92 | 0.81 | 0.00 | 0.00 | 0.00 | 0.76 | 2.49 | 2.14 |
| Indiana County, PA | 0.57 | 0.98 | 0.05 | 0.00 | 0.75 | 0.25 | 2.58 | 2.12 |
| Jefferson County, AL | 0.95 | 0.98 | 0.00 | 0.00 | 0.00 | 0.33 | 2.26 | 2.11 |
| Jefferson County, TX | 0.00 | 0.00 | 0.72 | 0.99 | 0.64 | 0.94 | 3.29 | 2.10 |
| Brazoria County, TX | 0.00 | 0.00 | 0.88 | 0.74 | 0.69 | 0.93 | 3.24 | 2.06 |
| Potter County, TX | 0.00 | 0.78 | 0.73 | 0.00 | 0.68 | 0.69 | 2.87 | 2.05 |
| Washington County, OH | 0.00 | 0.92 | 0.40 | 0.00 | 0.59 | 0.96 | 2.86 | 2.05 |
| Caddo Parish, LA | 0.00 | 0.00 | 0.88 | 0.53 | 0.98 | 0.89 | 3.28 | 2.05 |
| Ohio County, WV | 0.91 | 0.00 | 0.89 | 0.00 | 0.89 | 0.00 | 2.69 | 2.04 |
| Belmont County, OH | 0.33 | 0.61 | 0.75 | 0.00 | 1.00 | 0.00 | 2.69 | 2.03 |
| St. Charles Parish, LA | 0.00 | 0.00 | 0.70 | 0.89 | 0.58 | 0.99 | 3.16 | 2.01 |
| Monroe County, OH | 0.88 | 0.00 | 0.79 | 0.00 | 0.99 | 0.00 | 2.66 | 2.00 |
| Grimes County, TX | 0.00 | 0.63 | 0.68 | 0.00 | 0.70 | 0.88 | 2.89 | 1.99 |
| Kern County, CA | 0.00 | 0.00 | 0.99 | 0.32 | 0.89 | 0.98 | 3.19 | 1.98 |
| Pleasants County, WV | 0.00 | 0.91 | 0.42 | 0.00 | 0.70 | 0.68 | 2.71 | 1.97 |
| Marion County, WV | 0.97 | 0.43 | 0.13 | 0.00 | 0.85 | 0.00 | 2.37 | 1.95 |
| Choctaw County, MS | 0.76 | 0.66 | 0.00 | 0.00 | 0.00 | 0.97 | 2.38 | 1.94 |
| Plaquemines Parish, LA | 0.00 | 0.00 | 0.96 | 0.80 | 0.81 | 0.38 | 2.96 | 1.93 |
| Wayne County, MI | 0.00 | 0.90 | 0.00 | 0.71 | 0.00 | 0.93 | 2.55 | 1.93 |

The results of this exercise illustrate some of weaknesses of the aggregate approach. For example, Los Angeles County, CA ranks 15^th^ under this approach. Although the region is among the top counties for exposure to oil refining and natural gas-fired power generation, and it scores highly on measures of sensitivity due to health, environmental, housing, and other burdens, its large and diverse economy suggests that a shift away from fossil fuels is unlikely to pose serious long-term risks to the region’s economic vitality. By contrast, Kern County, CA ranks 35^th^ in the aggregate metric. Unlike Los Angeles County, Kern County is heavily reliant on fossil fuels—particularly oil—for local employment and tax revenue.^3,4^ This discrepancy highlights the notion that a single aggregate score—even one that takes into account the carbon intensity of the fuel—fails to capture the nuances that will vary considerably across energy types and communities.

References

1. White House Council on Environmental Quality. *Climate and Economic Justice Screening Tool: Technical Support Document Public Beta, Version 0.1*. https://static-data-screeningtool.geoplatform.gov/data-pipeline/data/score/downloadable/cejst_technical_support_document.pdf (2022).

2. U.S. Energy Information Administration. *Carbon Dioxide Emissions Coefficients*. https://www.eia.gov/environment/emissions/co2_vol_mass.php (2021).

3. Raimi, D. *et al.* *The Fiscal Implications of the US Transition Away from Fossil Fuels*. https://www.rff.org/publications/working-papers/the-fiscal-implications-of-the-us-transition-away-from-fossil-fuels/ (2022).

4. Raimi, D. *Mapping the US Energy Economy to Inform Transition Planning*. https://www.rff.org/publications/reports/mapping-the-us-energy-economy-to-inform-transition-planning/ (2021).
